# Supplementary material for: Initiating gender-affirming hormones for transgender and non-binary people: A qualitative study of providers’ perspectives on requiring mental health evaluations
Source: PLoS One. 2022 Aug 18;17(8):e0271785. doi: 10.1371/journal.pone.0271785 (PMC9387802; doi:10.1371/journal.pone.0271785)
Supplement: Attachment — Submitted filename: Response to Reviewers.docx [file pone.0271785.s001.docx]

**Comments to the Author**

1. Is the manuscript technically sound, and do the data support the conclusions?

The manuscript must describe a technically sound piece of scientific research with data that supports the conclusions. Experiments must have been conducted rigorously, with appropriate controls, replication, and sample sizes. The conclusions must be drawn appropriately based on the data presented.

Reviewer #1: Partly

Reviewer #2: Yes

2. Has the statistical analysis been performed appropriately and rigorously?

Reviewer #1: N/A

Reviewer #2: Yes

3. Have the authors made all data underlying the findings in their manuscript fully available?

The [PLOS Data policy](http://www.plosone.org/static/policies.action#sharing) requires authors to make all data underlying the findings described in their manuscript fully available without restriction, with rare exception (please refer to the Data Availability Statement in the manuscript PDF file). The data should be provided as part of the manuscript or its supporting information, or deposited to a public repository. For example, in addition to summary statistics, the data points behind means, medians and variance measures should be available. If there are restrictions on publicly sharing data—e.g. participant privacy or use of data from a third party—those must be specified.

Reviewer #1: No

Reviewer #2: Yes

4. Is the manuscript presented in an intelligible fashion and written in standard English.

PLOS ONE does not copyedit accepted manuscripts, so the language in submitted articles must be clear, correct, and unambiguous. Any typographical or grammatical errors should be corrected at revision, so please note any specific errors here.

Reviewer #1: No

Reviewer #2: Yes

5. Review Comments to the Author

Please use the space provided to explain your answers to the questions above. You may also include additional comments for the author, including concerns about dual publication, research ethics, or publication ethics. (Please upload your review as an attachment if it exceeds 20,000 characters)

Reviewer #1: I want to thank the authors for their work on this topic which is very relevant to both patients and providers vis-à-vis gender-affirming care. I appreciate the intent of the authors' research question here: "In this study, we aimed to describe the range of experiences and attitudes regarding hormone initiation among GAH providers and to assess factors that affect these attitudes, including the perceived advantages and disadvantages of each." In the abstract the aim is stated purely as descriptive, which—after reading the manuscript-- seems more accurate, less compelling or useful, and maybe even harmful.

The paper is largely descriptive and lacks a rigorous assessment piece without which I do not think the paper is publishable. I’m not sure if that is because the team is afraid of being critical of treatment approaches used by providers within their own social networks, or if it is fear of professional consequences for highlighting the lack of competence that comes with the gatekeeping model, or if the team really thinks these two models should be treated as equally legitimate approaches. Part of the problem might be that the analysis seems unfinished – I think you have some excellent quotes, organized into meaningful thematic sections, but the summaries of the data are lacking. Most of your paragraphs are largely quotes preceded by one sentence that summarizes them. I was wanting more from the authors in the results section in terms of moving beyond the quotes with synthesis and reconceptualization.

Consequently, the main finding -- that providers operate (or even "identify") on a continuum across these two “nonbinary” models -- is not terribly compelling. Models are just that – models/guideposts-- and not meant to be followed to the letter; they are meant to be adapted to contexts. So, the conclusion here that whispers at recognizing an unproblematic taxonomy of treatment approaches does not rigorously interpret the data that was provided. Maybe more importantly, the “treatment is not binary” feels a bit lazy and also degrades the idea of *gender* as something that is nonbinary and varies across time and space for individuals but also for societies, etc.

What might be helpful in the Discussion is to situate your findings within the literature on and the historical contexts of these models—where did they come from and why or in response to what? The idea presented in the intro (p. 4 line 75-76) that treatment approach is simply a matter of individual preference is not true and ignores not only institutional policies/mission&values but also each provider’s orientation to health justice/bodily autonomy, medical malpractice, social determinants of health, and the DSM and mental health, etc. What do your interviews say about what sustains these models or is informing their change/demise?

I also appreciate that in a couple places you gesture at what might be the key difference between these models: is it possible to “diagnose” someone’s gender in terms of exogenous hormone uptake? WPATH model says yes and it should be a MHP (there’s a history there that needs addressing), while informed consent says no – only the patient can really know what they might need. Just like some cis men ask for testosterone for a variety of reasons while some cis men choose other pathways for treatment, not all trans men want testosterone and some nonbinary AFAB persons also do. Cis men do not need a MHP to validate their request. Same with cisgender-affirming surgeries like facelifts and tummy tucks and a range of implants and other plastic surgeries.

**Author Response**: Many thanks to the reviewers for the engaged, thoughtful, and helpful comments. We have significantly revised the paper and have addressed their comments to the best of our ability. We agree that the more compelling of our findings is not the fact of continuum of care, but rather the factors affecting provider’s decisions on MHP involvement. We have reframed our paper and the discussion to center more explicitly on the differences in perception of transness. We highlight some of the extensive changes below.

“Two central questions guided providers’ processes for initiating GAH. The first is that of authority—who decides on a patient’s eligibility to initiate hormones. Providers relied less on MHPs when they leaned toward leaving this determination to the patient, trusting the patient’s own report of their transness as sufficient. Such an approach was common among providers who saw transness as an identity, or a self-understood phenomenon, rather than as a condition to be diagnosed (Fig 3). As such, the only authority on a person’s gender identity is the person themselves and mediation by an MPH is not only unnecessary, but potentially detrimental to their care. On the other hand, providers who understood transness through a medicalized lens tended to seek confirmation of eligibility, outsourcing that determination to an MHP. For them, a formal diagnosis of gender dysphoria, relegated to an MHP, was seen as part of a diagnostic process prior to treatment of a (mental health) condition; the patient’s self-determination was not sufficient (Fig 3). Additionally, in this group of providers, a narrow and (outdated) binary view of gender and transness was common and providers expressed misunderstanding of non-binary and genderqueer identities. Thus, the first central question differentiating the providers who follow WPATH versus those who use IC is whether the patient is to be trusted as best-positioned to report their own experience or whether the involvement of a MHP is needed.” *(Discussion Paragraph 1, Page 24)*

“The second question at the core of GAH initiation is that of the connection between mental illness and transness (Fig 4). While none of the providers who used the WPATH model explicitly described transness as a mental illness, the WPATH model is a medicalizing one; both the WPATH organization and the Standards of Care were developed over the past near century—primarily by MHPs, who understood transness as a “deviance” or “pathology” [35]. Providers who followed the WPATH model were persistently concerned about mental illness, “destabilization,” and “serious issues” among their transgender patients with GAH initiation, occasionally based on anecdotal experience, but not supported by the literature. These concerns lead them to view the entire initial interaction with their transgender patients through the lens of mental health. They then relegated the decision on GAH initiation to an MHP and were more likely to require a letter (Fig 4).” *(Page 25, Paragraph 1)*

“Providers relied less on MHPs when they saw severe mental health disorders as outliers rather than the norm among transgender people and felt comfortable screening for them. Additionally, there seemed to be a difference between the provider groups in the anticipated effects of GAH on existing mental health. In the group that used an IC model, several providers noted the possibility of improvement of mental health with hormones [24,26,28,29], or were not concerned about a patient’s ability to adhere to the hormones; rather, they focused on improving access to GAH and removal of any potential barriers, including letter requirements. In the group that required letters, providers voiced a concern about mental health deterioration following hormone initiation (Fig 4). Overall, providers who expressed a perception of initiating GAH as a process no different from other healthcare were more likely to use a direct approach to prescribing GAH with fewer requirements.” *(Pages 25-26, Paragraph 2)*

Here are some more specific comments on the manuscript:

Results:

P. 10 Lines 165-170 Your paragraph on provider decision-making is a very good example of the problem with the results section. First, I cannot tell what “decision-making” (165) even refers to. Is it whether to send them to an MHP, whether to treat them at all, whether to prescribe hormones? And “decision-making for a variety of factors” is incredibly vague, providing no framework for the reader. Also, you should avoid requiring the reader to read several quotes in the mid-sentence bracketed by parentheses – it is really hard to follow. In line 170 you need to specify that you are talking about *hormonal* transition – or if you are talking about some other transition, say so, and say why these providers are concerned with that. This paragraph provides evidence of a wide range of responses but no synthesis that follows or reconceptualization. What does it mean that a provider bases their prescribing on whether a patient has insurance while another cares about whether or not they are out? These are really big statements that are left behind with no scaffolding from the author team.

**Author Response:** Thank you for helping us clarify this introductory paragraph. We have made the below changes to the paragraph and attempted to clarify that further analysis is to follow.

“Providers attributed their decision about MHP involvement to a variety of factors. Themes ranged from perceptions regarding practical concerns with individual patients, to broader attitudes regarding gender identity (“Are they insured or not? Whose insurance are they on? Are they out? … I think the biggest barrier is the financial piece and the safety and security”); and broader attitudes regarding gender identity (“Some people do have gender dysphoria but not everybody is dysphoric about just being who they are”; “I think we are sometimes too rigid about how we define gender”). All providers considered the social challenges related to both social and medical transition. They acknowledged the potential social, emotional, and financial implications of potential rejections and importance of social networks in supporting a trans person as they transitioned. These themes were present in our interviews with providers regardless of their approach to hormone initiation. However, there were some distinct differences by approach. In the following sections we review the main themes identified and analyze them according to provider approach to MHP involvement.” *(Page 12, Paragraph 1)*

173-175

I don’t think a paragraph can consist of only one sentence, a problem that appears in other parts of this paper. (This sentence is poorly written too – “the need” appears twice.) Can you say more about what this section is doing? Give the reader more of a framework from which to interpret the section. Please eliminate/build out all one-sentence paragraphs.

**Author Response:** Thank you for helping us improve the clarity of our paper. We have rephrased this paragraph as follows:

“In the following section we present the themes among providers who strictly required an MHP evaluation prior to GAH initiation. Within this group, we identify the following themes: a) the importance of MHP consultation to ascertain a diagnosis of gender dysphoria; b) concern about unstable or undetected mental health issues; and c) the provider’s own confidence in addressing these issues. We detail each of these themes below. These and other advantages and disadvantages of involving an MHP are summarized in Fig 2.” *(Pages 12-13, Paragraph 2)*

Furthermore, we have eliminated one sentence paragraphs through the remainder of the paper.

178-79

This sentence seems to contradict itself: “While some who required a mental health evaluation acknowledged that they ultimately rely on a patient’s self-identification as transgender.” How is it possible to claim that they really rely on the patient’s self-id when they in fact are relying on an MHP as a rule? You need to elaborate on what they were saying – maybe that they want to ensure that the patient is resilient enough to endure the social upheaval that might emerge? -- or make note of this glaring contradiction. When the author team does not either explain the quote further and/or make note of these contradictions, it is almost like a defense of this model or approach coming through. Similarly, when providers are saying for example: 1) they do not understand nonbinary identities and so they require an MHP, or 2) they got burned when two of their patients killed themselves and so they send all patients to an MHP, the author team should further elaborate on what these explanations suggest about the model they are using and about their approach/view of patients whom they are treating.

**Author Response:** We have added the following explanations to these particular instances, as follows:

“This provider acknowledges that in principle, they agree that transness is best attested by the person themselves. Yet, there is an apparent discrepancy between that claim and the provider’s insistence on having that identity verified by an MHP. Most physicians will not explicitly dismiss a patient’s gender identity, likely because of social desirability bias. However, ultimately, it is disbelief or doubt in the patient’s transness that determines the course of action.” *(Pages 13-14, Paragraph 3)*

Regarding nonbinary identities, we have added the following text:

“Both providers (#14 and #15) display fundamental misunderstandings of non-binary and genderqueer identities or of ways of being trans that didn’t match up with their expectations about sex/gender. Providers were confused when patients with such identities did not fit the providers’ binary understandings of male/man or female/woman identities. Provider #15 fails to recognize the validity of non-binary and genderqueer identities—comparing and contrasting them to intersex people, while pathologizing the latter. As such, when a patient’s goal is that of a non-binary or genderqueer presentation, the provider thinks this is effectively a request to become intersex. This conflation suggests both the psychiatric pathologization of non-binary identity (as the patient did not have a “real hormonal problem”) and dismissal of non-binary or genderqueer identity (“couldn’t contribute to that”). The provider therefore feels compelled to resort to psychiatric evaluation and treatment.” *(Pages 14-15, 3)*

Following the quote from the physician who reports being burned by suicidal patients, we further explain:

“This provider had two traumatic experiences with patients. Their position is now to require letters before starting hormones—suggesting a view that mental health problems need to be addressed prior to GAH treatment despite the known benefits of GAH. If the concern is that mental health problems may be missed without the letter requirement, why not start hormones and encourage patients to see an MHP concurrently? There is an unsubstantiated sense that hormones themselves might cause ill effects on mental health, and that a letter is an adequate and sufficient way to ensure that such effects are addressed. *(Page 16, Paragraph 3)*

p.14, 267

Section on MHP availability and expertise is poorly organized and/or written. The paragraph leads with a very important observation, but the rest of the first paragraph is one quote. My rule of thumb is to *never* end a paragraph with a quote, whereas most paragraphs in your Results section end with a quote and simply move on to the next theme. The data needs restating/summarizing/linking/synthesis that nods at your broader argument or toward what is coming in the next section, etc. The Results is very choppy as currently written.

**Author Response:** We have restructured the results section, including reordering of several sub-sections, and ensured summary and further analysis following quotes. We hope that this makes for a smoother reading experience and interesting analysis.

p. 15, 314-333

Same concerns here as preceding comment. This section is trying to do way too much without providing clear structure, clear writing, and clear scaffolding/framing for the reader to understand what to do with the data. It consists of 5 quotes and 2.5 sentences of your own – a “healthy” paragraph might have four sentences and one or two quotes.

**Author Response:** We have added significant summary and analysis for this section as well.

p. 17, 350-353

Provider #17 appears in the section about strict requirement of MHP (p.11, line 181). In this paragraph they are referred to as a provider who does NOT universally send to an MHP.

**Author Response:** We have addressed this and changed this paragraph.

There are lots of rich data points in this manuscript, but I think the team needs to figure out what is the most compelling finding and completely rewrite the Discussion section. What can you say about these models and the interests they serve? I encourage the team to comb through each paragraph in the Results and rewrite it with stronger frameworks and syntheses that generate a more coherent critique and move the literature forward. A section that felt absent, that may help you critique the models more rigorously, is some kind of synopsis about how these providers came to care for trans/nonbinary patients (i.e. one of your interview domains to explore). What was it about their training? What was lacking or what informed it that brought them to these different approaches? I will say, for example, that very well-meaning providers/colleagues, who use the WPATH model, still think that using the “genderbread man” or the “gender unicorn” is a useful pedagogical tool to teach family medicine residents about gender-affirming care. What does that say about the level of medical sophistication around gender and gender-affirming care? I think the lack of non-White – and specifically Black providers – also limits your takeaways. I don’t know any Black providers who are members of WPATH or who use the WPATH model. Why do you think that is? Well, you cannot say because you don’t have the data, but you might check the literature on this/the history of racism in medicine and mental healthcare/the high cost of WPATH membership/the fact that many Black providers who do trans care do so at safety net clinics and health centers.

**Author Response:** We are very grateful to the reviewer for these insightful comments. We hope that we have managed to address them by significantly restructuring and rewriting both the results and the discussion sections.

While our interviews contained data about the paths providers took to become involved in hormone prescription, our findings do not allow us to make broad categorizations or come to significant conclusions regarding patterns in training and their effect on provider approach to hormone prescription.

We could not agree with you more regarding both the lack of diversity in medicine, and the “flatness” and lack of nuance in medical training - not solely regarding gender and sexuality, but more broadly as it relates to many social questions, including racism in medicine and in society at large.

There are many places where citations are missing. This paper cites literature very sparsely. There is a rich literature on these models and on the associations between GAH uptake and mental health benefits, but I do not see much of them cited. Similarly, there is literature on reasons why patients might not disclose gender identities fully to their providers, particularly nonbinary patients, but these studies don’t seem to be cited or used to advance an argument/critique either. Another rich literature to draw upon is that of medical education and training (or lack thereof or inadequacy of). I think this paper can be very rich and make a valuable contribution, but in its current form, it does not.

**Author Response**: Thank you for drawing our attention to this. We have thickened both our analyses and our citations and aimed to base our argumentation on existing literature.

Reviewer #2: This is an extremely well written and important manuscript. These data are nuanced and complex and will add a missing component to the dearth of research on provider practice and motivation around gender-affirming hormone care. Well done.

6. PLOS authors have the option to publish the peer review history of their article ([what does this mean?](https://journals.plos.org/plosone/s/editorial-and-peer-review-process#loc-peer-review-history)). If published, this will include your full peer review and any attached files.

If you choose “no”, your identity will remain anonymous but your review may still be made public.

**Do you want your identity to be public for this peer review?** For information about this choice, including consent withdrawal, please see our [Privacy Policy](https://www.plos.org/privacy-policy).

Reviewer #1: No

Reviewer #2: No

[NOTE: If reviewer comments were submitted as an attachment file, they will be attached to this email and accessible via the submission site. Please log into your account, locate the manuscript record, and check for the action link "View Attachments". If this link does not appear, there are no attachment files.]

While revising your submission, please upload your figure files to the Preflight Analysis and Conversion Engine (PACE) digital diagnostic tool, <https://pacev2.apexcovantage.com/>. PACE helps ensure that figures meet PLOS requirements. To use PACE, you must first register as a user. Registration is free. Then, login and navigate to the UPLOAD tab, where you will find detailed instructions on how to use the tool. If you encounter any issues or have any questions when using PACE, please email PLOS at figures@plos.org. Please note that Supporting Information files do not need this step.
