# Supplementary material for: Initiating gender-affirming hormones for transgender and non-binary people: A qualitative study of providers’ perspectives on requiring mental health evaluations
Source: PLoS One. 2022 Aug 18;17(8):e0271785. doi: 10.1371/journal.pone.0271785 (PMC9387802; doi:10.1371/journal.pone.0271785)
Supplement: Attachment — Submitted filename: Response to reviewers.docx [file pone.0271785.s002.docx]

Response to the Editor and reviewers:

Author Response: Many thanks for your feedback. Our responses to your thoughtful comments and suggestions are below.

First, it must be said that the qualitative methodology is very well used in terms of description of the procedures and the reporting of the results.

My concern is with the conclusions. I feel that the first two paragraphs of the conclusion should be followed by the last paragraph of the Continuum section.

Author Response: We have changed the manuscript based on your helpful comments. We moved part of the last paragraph from the Continuum section to an independent Limitations section (based on recommendations by Reviewer #1) and we moved the second part of that paragraph to follow the first two paragraphs of the conclusion.

The last 3 paragraphs of the conclusion slightly extrapolate the objectives and results of the study and seem to me to make strong statements about possible consequences for the collective and individual health of trans people, but which go beyond the present article, which is exploratory in nature.

These paragraphs and also the conclusion of the abstract are categorical as if they came from a research of a descriptive/quantitative nature and deserve more nuance.

Author Response: We appreciate this insight, and have revised our conclusion to more carefully reflect the findings of our study, and the inability to generalize from our study to the general “provider” population. We hope that the more nuanced phrasing captures both the nature of our findings, the insights and limitations of qualitative research, and potential implications of our findings on the field at large.

Furthermore, I feel that the discussion and conclusion should focus on the US context as this issue is more acute in the context in which the research was situated and is present in a different way in other parts of the globe.

Author Response: We added an acknowledgement of the local context to the limitations section (“while the WPATH guidelines are international, our study was limited to providers in the United States, and may not reflect practice patterns in other countries.”), as well as explicitly stating it in the abstract.

[Note: HTML markup is below. Please do not edit.]

Reviewers' comments:

Reviewer's Responses to Questions

**Comments to the Author**

1. If the authors have adequately addressed your comments raised in a previous round of review and you feel that this manuscript is now acceptable for publication, you may indicate that here to bypass the “Comments to the Author” section, enter your conflict of interest statement in the “Confidential to Editor” section, and submit your "Accept" recommendation.

Reviewer #1: (No Response)

Reviewer #2: All comments have been addressed

Reviewer #3: All comments have been addressed

2. Is the manuscript technically sound, and do the data support the conclusions?

The manuscript must describe a technically sound piece of scientific research with data that supports the conclusions. Experiments must have been conducted rigorously, with appropriate controls, replication, and sample sizes. The conclusions must be drawn appropriately based on the data presented.

Reviewer #1: Partly

Reviewer #2: Yes

Reviewer #3: Yes

3. Has the statistical analysis been performed appropriately and rigorously?

Reviewer #1: N/A

Reviewer #2: Yes

Reviewer #3: Yes

4. Have the authors made all data underlying the findings in their manuscript fully available?

The [PLOS Data policy](http://www.plosone.org/static/policies.action#sharing) requires authors to make all data underlying the findings described in their manuscript fully available without restriction, with rare exception (please refer to the Data Availability Statement in the manuscript PDF file). The data should be provided as part of the manuscript or its supporting information, or deposited to a public repository. For example, in addition to summary statistics, the data points behind means, medians and variance measures should be available. If there are restrictions on publicly sharing data—e.g. participant privacy or use of data from a third party—those must be specified.

Reviewer #1: No

Reviewer #2: Yes

Reviewer #3: Yes

5. Is the manuscript presented in an intelligible fashion and written in standard English?

PLOS ONE does not copyedit accepted manuscripts, so the language in submitted articles must be clear, correct, and unambiguous. Any typographical or grammatical errors should be corrected at revision, so please note any specific errors here.

Reviewer #1: Yes

Reviewer #2: Yes

Reviewer #3: No

6. Review Comments to the Author

Please use the space provided to explain your answers to the questions above. You may also include additional comments for the author, including concerns about dual publication, research ethics, or publication ethics. (Please upload your review as an attachment if it exceeds 20,000 characters)

Reviewer #1: Thank you for the opportunity to read the revised version of the manuscript. It is a much stronger, coherent, and well-written paper. There are minor, albeit fairly fundamental, revisions I would suggest making.

1) There remains conceptual murkiness around the fundamental concept discussed in this paper - gender identity - or maybe just identity itself. First, the last sentence of the intro where "transgender" is defined is not useful at all. It's circular and also just incorrect. What are non-cisgender identities? For example, trans AND cis adults alike identify as men or as women. Please provide a more nuanced and careful definition.

Author Response: We agree with the reviewer that trans and cis individuals can identify as men or women. We intend this sentence not as a definition, which we believe at this point is basic and familiar enough to the average reader of this paper as to not require a definition, but rather as a clarification that we use the term in a non-exclusive way. We have added clarification that we use the term as an umbrella term, including non-binary and genderqueer individuals among others.

Relatedly, on the next page, in methods, some clarifications may be helpful. I'm not sure what the reflexivity section is trying to do. The second sentence is very confusing - either say your team consists of "cisgender or non-binary as well as straight and queer" individuals or say it consists of "a diversity of genders and sexualities". As it currently stands I'm not sure what you are trying to say about cisgender, queer, and non-binary people on your team that is unique from the diversity of sexualities and genders. Is queer a gender? Are non-binary people asexual? Are cisgender people straight? Why have those three been singled out from the diversity? Further there seems to be a sentence missing about why it's important to note that no one on the author team has ANY experience with seeking medical transition and how that may impact the findings in this paper. Stating your privilege as researchers would remain true had you included a co-author with this lived experience.

Author Response:

We have expanded on our experiences with medical transition:

“While some of us have sought a variety of forms of medical gender affirmation (in addition to the casual, everyday gender affirmation that cisgender people receive by society at large), none of us have accessed gender-affirming hormones. This latter fact may well have impacted our understanding of the process and our interpretations”

Given our specific identities, our reflexivity statement as it currently stands is the most precise and concise way to share with the reader how our identities might affect our perspectives. To answer the reader’s specific questions, for some of us Queer is a gender identity, for others it is a sexual identity. Regardless of our identities along the cis/trans spectrum, some identify as men, some as women (and some, nonbinary or queer) – thus the diversity of genders is distinct from whether or not we are cis or trans. We found it important to share the diversity of identities in relation to cis/transness in this context, independent of our genders (man, woman, non-binary, etc.). Those specific identities mentioned were singled out, because those are our identities.

Presumably the reader knows that non-binary people can have any sexuality, as can cisgender people. Agreed that stating our privilege is true regardless of our gendered lived experiences.

In the next paragraph, purposive sampling was used to recruit providers who "self-identify" as using WPATH or IC. That term is really confusing first of all. To my knowledge, providers do not conflate their treatment model preferences with self-identification. Secondly, it doesn't seem like that inclusion criteria is true. Table 1 says that 17% of your sample uses "other" as their treatment model/guidance. Please align your methods and inclusion criteria with your results.

Author Response: We replaced the words self-identify here with self- described. Our purposive sampling was meant to ensure that we have representation of providers from both groups; identifying as “other” was not an exclusion criterion. Hence both statements (in the introduction and in the table) are correct.

2) Page 12/line 188-190 there is a redundant theme stated about "broader attitudes regarding gender identity" with very different quotes as examples that follow this same theme - please clarify what is different about these same themes by way of these quotes.

Author Response: Thank you for noticing this error – this was a redundancy in text that was corrected

3) Transsexual is spelled differently in different parts of the paper. Technically, it has two "s"s but some adhere to "transexual" (i.e. not unlike nonbinary vs. non-binary). Please choose which version you are meaning to use and stick with it.

Author Response: We use the term “transsexual” only in citation of other sources, and follow the spelling in the titles as published. We have corrected the spelling where it appears in the body of the text referring to DSM-III.

4) The quote from #16/line 391 should be modified or commented upon. Why did you think it is important to include a quote, referred to as "their insight," where an ostensible ally is saying that many seem to think this provider has "had more of the Kool-Aid" because they do not gatekeep? Please interpret or share the importance with the reader or don't include it. As it stands, inclusion of this analogy perpetuates the idea of a growing trans medical industrial complex as driven by a cult like that of the Jim Jones kind where it ends in mass suicide for mostly black Americans. Do you think the provider's ideas have "evolved" as you say prior to the quote? I think they sound ambivalent/conflicted about their choices and/or unfairly persecuted by acting as an ally. Maybe say more about what is going on here; otherwise I would not include the "Kool-Aid" comment as an insight around an evolution of ideas.

Author Response: Thank you for the encouragement to further explain this quote (especially in light of the participant’s use of an expression with violent origins and negative contemporary connotations). We have added the following interpretation of the quote:

“This provider describes their own learning process, with increased understanding of gender as a continuous, social construct. Their newfound understanding, which they acknowledge others might view as ideologic following, has encouraged them to move their practice away from the WPATH model and toward IC.”

5) Granted i have not had time to do as careful a read of the discussion as I'd like, it seems much stronger and coherent and comprehensive than the initial version (the "Continuum" subheader may need something more, and you might add a "Limitations" subheader which currently falls under "Continuum". Thank you so much for the thoughtful revision.

Author Response: Thank you so much for all your helpful comments.

We have added a Limitations paragraph to the end of the Discussion section. While more detail could be added to the Continuum section, we agree with the reviewer comments from the first R&R, that this is not the most important finding of the paper. Thus, we have elected not to expand on this further so as not to distract from the main purposes of the manuscript.

Reviewer #2: (No Response)

Reviewer #3: This is an important topic, and I appreciate what your qualitative study is able to add to the discussion around MHP letter requirements before initiation of gender-affirming care.

I said "no" for number 5 because some of the additions made after the first round of reviews disrupted the flow of the manuscript.

While commenting on and contextualizing the quotes is important, the placement of your critique immediately after the quotes in the results (for example, in lines 229-232) made me feel the opinion of the authors in a way that was distracting as a reader - even though I agree with your interpretation/critique/additions.

Author Response: Thank you for pointing out the excessive opinion here, rather than straightforward interpretation. We have revised the interpretation following the quote here to state “Yet, there is an apparent discrepancy between that claim and the provider’s insistence on having the patient’s identity verified by an MHP. Despite their stated agreement with the principle of believing a patient about their identity, ultimately, it is disbelief or doubt in the patient’s transness that determines this provider’s course of action”

To back up your critiques of the quotes of the respondents who required letters, you included many more citations in this draft, which was important. Yet again, I think that discussing the quote within the context of available literature (for example, lines 275-285) would have been more appropriate in the discussion section.

Author Response: Agreed! We moved this section to the discussion, which hopefully also improves the flow.

In fact, I found myself wondering who providers 13-15 were, and getting upset that they feel qualified to do gender-affirming care. Perhaps this was the intent, but it made it difficult for me to maintain attention through the rest of the manuscript. And, as someone who has been quoted out of context as a participant in a manuscript on a different topic before, I couldn't help but hope those participants will not ever be able to identify themselves in your manuscript (and that they attend trainings by folx with lived experience).

Author Response: Sadly, we agree. We understand that they may be upset if they recognize themselves in the manuscript, and may feel misunderstood; it is our interpretation that they do not necessarily have full awareness of the meaning of some of their statements, hence may feel misquoted. Nevertheless, having discussed this within the research team extensively, we ultimately feel that these are the best interpretations of our data, and that we are giving an honest account of some of the issues in the field

Line 214, please make sure it says MHP, not MPH.

Author Response: Corrected, thank you.

I appreciate this work and otherwise found the manuscript compelling and well-written. Thank you for doing this study!

7. PLOS authors have the option to publish the peer review history of their article ([what does this mean?](https://journals.plos.org/plosone/s/editorial-and-peer-review-process#loc-peer-review-history)). If published, this will include your full peer review and any attached files.


If you choose “no”, your identity will remain anonymous but your review may still be made public.


**Do you want your identity to be public for this peer review?** For information about this choice, including consent withdrawal, please see our [Privacy Policy](https://www.plos.org/privacy-policy).

Reviewer #1: No

Reviewer #2: No

Reviewer #3: No

[NOTE: If reviewer comments were submitted as an attachment file, they will be attached to this email and accessible via the submission site. Please log into your account, locate the manuscript record, and check for the action link "View Attachments". If this link does not appear, there are no attachment files.]

While revising your submission, please upload your figure files to the Preflight Analysis and Conversion Engine (PACE) digital diagnostic tool, <https://pacev2.apexcovantage.com/>. PACE helps ensure that figures meet PLOS requirements. To use PACE, you must first register as a user. Registration is free. Then, login and navigate to the UPLOAD tab, where you will find detailed instructions on how to use the tool. If you encounter any issues or have any questions when using PACE, please email PLOS at [figures@plos.org](mailto:figures@plos.org). Please note that Supporting Information files do not need this step.
